# Exploration of Reactive Black 5 Dye Desorption from Composite Hydrogel Beads—Adsorbent Reusability, Kinetic and Equilibrium Isotherms

**DOI:** 10.3390/gels9040299

**Published:** 2023-04-03

**Authors:** Cristina-Gabriela Grigoraș, Andrei-Ionuț Simion, Lidia Favier

**Affiliations:** 1 Department of Food and Chemical Engineering, Faculty of Engineering, “Vasile Alecsandri” University of Bacău, Calea Mărășești 157, 600115 Bacău, Romania; 2 Ecole Nationale Supérieure de Chimie de Rennes, University Rennes, CNRS, ISCR–UMR6226, F-35000 Rennes, France

**Keywords:** cherry stones, chitosan, Reactive Black 5, hydrogel beads, desorption, Box–Behnken Design, kinetics, equilibrium isotherms

## Abstract

A low-cost adsorbent was prepared by using cherry stones powder and chitosan and used to retain Reactive Black 5 dye from aqueous solution. Then, the spent material was submitted to a regeneration process. Five different eluents (water, sodium hydroxide, hydrochloric acid, sodium chloride and ethanol) were tested. Among them, sodium hydroxide was selected for an advanced investigation. Values of three working conditions, namely the eluent volume, its concentration and the desorption temperature, were optimized by Response Surface Methodology-Box–Behnken Design. In the established settings (NaOH volume: 30 mL, NaOH concentration: 1.5 M, working temperature: 40 °C), three successive cycles of adsorption/desorption were conducted. The analysis performed by Scanning Electron Microscopy and by Fourier Transform Infrared Spectroscopy revealed the evolution of the adsorbent throughout the dye elution from the material. Pseudo-second-order kinetic model and Freundlich equilibrium isotherm were able to accurately describe the desorption process. Based on the acquired results, our outcomes sustain the suitability of the synthesized material as dye adsorbent and the possibility of efficaciously recycling and reusing it.

## 1. Introduction

Sustainable development is one of the major contemporary concepts frequently involving the increasingly common use of resources that can be regenerated and the intensive preferences for environment-friendly products. For its implementation, continuous efforts are made all over the world in different sectors of activity [1].

In the treatment process of wastewater contaminated with various emerging pollutants (dyes, pharmaceuticals, personal care products, pesticides and so on), adsorption is among the prevalent applied technologies [2,3,4,5,6,7]. It implies the contact between the polluted aqueous media with the surface of a certain material (the adsorbent) having the ability of retaining the pollutants (the adsorbates) and the transfer of the respective pollutants from water to the adsorbent. In comparison with other chemical, biological or physical methods connected to water depollution, adsorption has several advantages including reduced cost, easy operation, absence of unwanted by-products or possibility of using a wide variety of adsorbents going from cationized cellulose [8] or composites containing industrial wastes [9,10] to those based on various oxides [11] or on functionalized nanoparticles [12,13,14]. These materials are often highly efficient in removing water noxious compounds, but due to the limited number of sites available, in time, they become saturated, this fact representing a restrictive factor and the foremost adsorption drawback.

Even though there are studies indicating that the exhausted adsorbents can be transformed in new value-added products such as fertilizers, catalysts, additives etc. [15], their recycling in order to serve the initial purpose remains a real challenge. The regeneration process can be accomplished by multiple methods such as thermal treatments [16], microwave heating [17], electrochemical treatments [18] etc. Despite their recognized effectiveness, the aforesaid methods imply preliminary steps and elevated costs and are susceptible to release dangerous products. An attractive alternative consists in desorbing the retained contaminants into an adequate solvent and reusing the recovered adsorbent.

The current work is a continuation of some of our results previously published [19]. In the anterior paper, we entrapped powder of cherry stones (an undervalued by-product from the cherry processing industry) on the natural polymeric matrix of chitosan. The obtained material was used to retain azo dyes from water. One of the model molecules was represented by Reactive Black 5 (RB). This pollutant is frequently encountered in textiles coloring, and it is highly soluble in water as well as a harmful compound answerable for carcinogenic, mutagenic and toxic effects on humans and the environment [20,21,22]. Some of the parameters affecting the process were optimized, and kinetic and equilibrium isotherm models were applied to the collected data.

Although the adsorption is one of the most employed methods for removal of refractory compounds from water, often the desorption is analyzed only from the point of view of the number of cycles of using the adsorbent material. There are not many systematic studies concentrated on establishing the kinetic and equilibrium isotherm models, describing the desorption process even less from such a material as the one reported here.

With this background and with the desideratum of offering relevant information on the desorption field, we developed at this point an experimental program with Reactive Black 5 as an example molecule.

In a first step, a screening of desorption solvents was conducted in order to establish the most appropriate one. Secondly, an optimization process was carried out via a Response Surface Methodology-Box–Behnken Design (RSM-BBD). Solvent volume, its concentration and working temperature were the input parameters, while the desorption efficiency was chosen as output function. In the third step, several cycles of adsorption–desorption were led. In parallel, scanning electron microscopy (SEM) and Fourier-transform infrared spectroscopy (FTIR) were used to analyze the prepared adsorbent after each cycle. Lastly, various kinetic and equilibrium isotherm models were applied to investigate the RB elution from the adsorbent.

## 2. Results and Discussion

### 2.1. Optimization of Reactive Black 5 Dye Desorption by Response Surface Methodology

Prior to optimization, two different steps were conducted.

In the first one, RB adsorption was piloted. The utilized conditions were adjusted in one of our precedent works [19]. Briefly, in 5 different runs, 30 mL of pollutant aqueous solution having a concentration of 30 mg/L were put in contact with 3 g of the prepared adsorbent for a period of 6 h at a temperature of 30 °C. A removal efficiency of 91.12 ± 1.06% and an adsorption capacity of 0.273 ± 0.022 mg/g were recorded.

In the second phase, 3 g of loaded adsorbent was immersed in 30 mL of various eluents and kept at 30 °C for 6 h.

Distilled water, one base (sodium hydroxide, 1 M), one acid (hydrochloric acid, 1 M), a salt (sodium chloride, 1 M) and an alcohol (ethanol, 1 M) were chosen as possible desorbents. The selection was made taking into consideration the ability of eluting the contaminant and the possible impact on the environment and human health. It must be mentioned that Australian National Industrial Chemicals Notification and Assessment Scheme using the Inventory Multitiered Assessment and Prioritisation (IMAP) categorizes chemicals into three classes: Tier I which includes compounds dissociable in environment; Tier II with compounds that can be dangerous and need additional exploration; and Tier III which comprises compounds that require detailed investigation of the aspects identified in Tier II. Therefore, due to the fact that they do not represent an extreme threat neither to the environment nor to humans, Tier I chemicals are recommended to use.

According to IMAP, all five tested eluents of this study are in Tier I in terms of environment safety. With regard to human safety, water, sodium chloride and ethanol are Tier I, sodium hydroxide is in Tier II, and hydrochloric acid is in Tier III [23].

**Figure 1:**
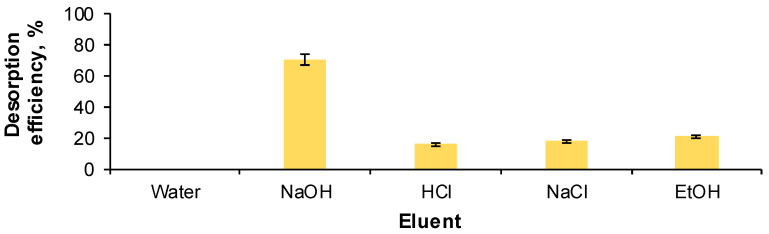
Impact of eluent type on the desorption efficiency of Reactive Black 5 dye from the prepared adsorbent (RB volume: 30 mL, RB initial concentration: 30 mg/L, eluent volume: 30 mL, eluent concentration: 1 M, adsorbent amount: 3 g, contact time: 6 h, temperature: 30 °C).

As can be seen in Figure 1, when water was used as the control eluent, no desorption was recorded.

As expected, since RB has an acidic nature, the desorption efficiency was reduced when HCl was used as the desorbent. A low desorption was also observed in the case of NaCl and ethanol (below 20%). On the contrary, when NaOH was used, RB manifested attraction to the alkaline media, the desorption efficiency being of 70.137 ± 0.224%. Therefore, the eluent screening conducted to the conclusion that sodium hydroxide is appropriate for the regeneration of the synthesized material.

For the optimization of the desorption process, a Response Surface Methodology (RSM) with Box–Behnken Design (BBD) based on three-level partial factorial design [24] was applied and served to estimate the parameters of a quadratic model.

A matrix of 15 runs with three central points and three factors (sodium hydroxide volume—A; sodium hydroxide concentration—B; and working temperature—C) was developed as presented in Table 1.

**Figure 2:**
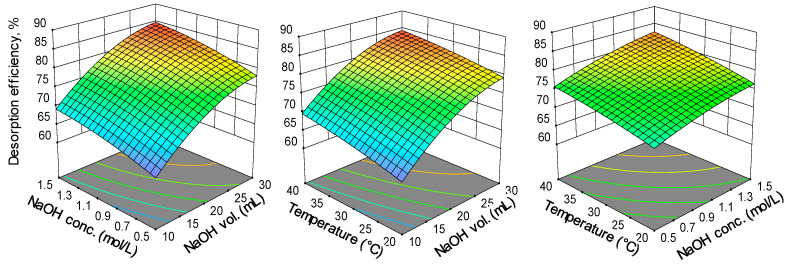
3D response surface plots of Box–Behnken Design.

The results obtained by RSM-BBD (Figure 2) show that an augmentation of the values of all three parameters involved in the experimental program is beneficial for the desorption efficiency of RB dye from the loaded adsorbent.

A higher volume of desorbent insures a better interaction between the contaminant from the solid phase and the eluent from the aqueous media. The same explanation was published by Daneshvar et al. [25]. According to their results, obtained for the desorption of methylene blue from an alga mass, the impact of eluent volume is critical since, up to a certain limit, its increase expands the removal of the dye from the adsorbent.

The raise of NaOH concentration from 0.5 M to 1.5 M changes the pH of the desorption environment and acts in favor of releasing the retained dye from the adsorbent. Hsueh et al. [26] investigated the desorption of RB from an activated alumina-supported iron oxide-composite used as catalyst for a Fenton degradation and revealed that sodium hydroxide in a solution with pH 12 is able to desorb almost completely the dye even after three cycles of adsorption–desorption. On the other hand, in their exploration of desorbing Congo red dye from crosslinked cellulose–chitosan foam, Kim et al. [27] discovered that a too high medium strength caused by elevating the NaOH concentration can be detrimental for the desorption.

Temperature also improves the solubility and the migration of RB from the synthesized material, enhancing its recovery as stated also by other studies aiming to recuperate dyes from various adsorbents [28,29]. At the same time, even though temperature provokes a swelling effect on the internal structure of the materials which ameliorates the migration of the contaminant to the liquid phase, its value must be carefully controlled because an excessive distention can be also responsible for unwanted ruptures and denaturation and even for an irreversible degradation of the adsorbent.

The aim of the RSM-BBD optimization was to attain the highest desorption efficiency. The expression of the mathematical model is given by the Equation (1).

(1) $$D,\;\%=77.770+7.650\times A+3.190\times B+2.800\times C+0.116\times A\times B-0.295\times A\times C++0.271\times B\times C\;-3.110\times A^{2}-0.876\times B^{2}-0.476\times C^{2}$$

The reliant significance of the terms is determined by the coefficient signs: the positive ones have a combined effect and intensify the response function with their increase while the negative ones suggest that the parameter impacts the response function by decreasing it.

**Figure 3:**
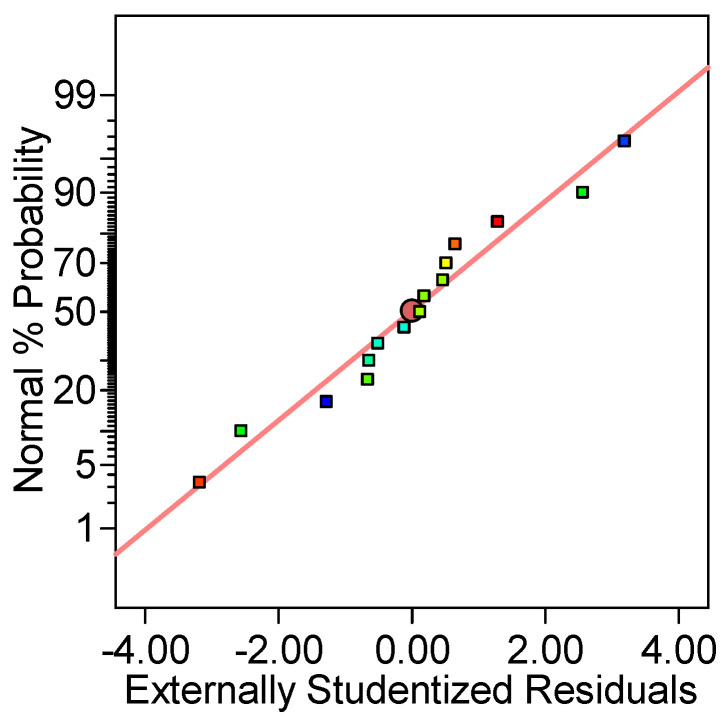
The Normal probability plot of residuals obtained for RB desorption efficiency.

**Figure 4:**
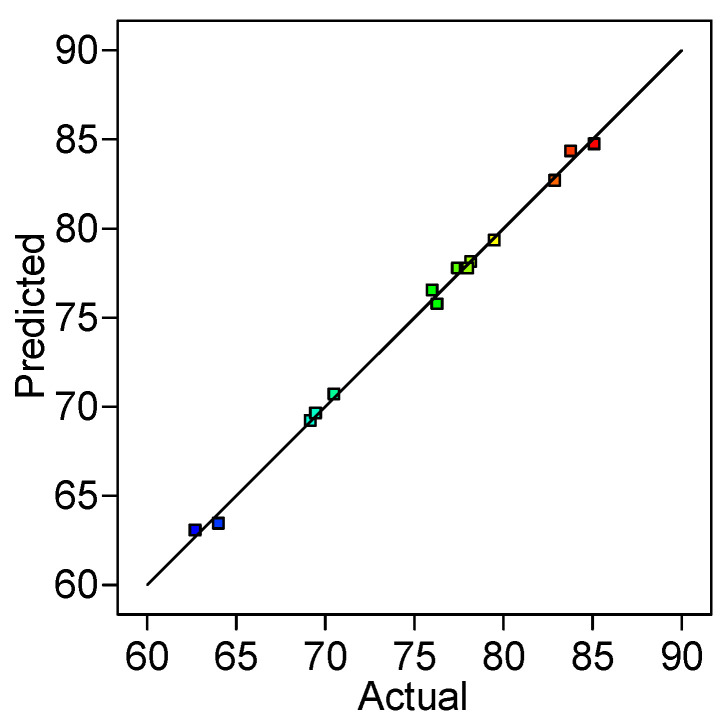
Experimental versus predicted values of RB desorption efficiency.

From Figure 3, it can be noted that almost all points follow a straight line, sustaining that the data have a normal probability. Then again, the representation exposed in Figure 4, discloses a linear distribution and points out a reasonable connection between the experimental and the predicted values.

**Figure 5:**
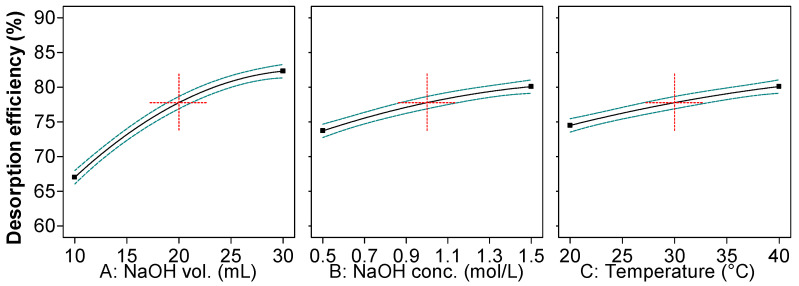
Impact of the parameters affecting the desorption of Reactive Black 5 dye from the prepared adsorbent.

The illustrations in Figure 5 and the data given in Table 2 detail the results of the analysis of variance for the parameters evaluated in RB desorption. The model *F*-value of 201.82 implies that this is significant, and that there is only 0.01% chances that a *F*-value this large could occur due to noise. The *p*-value inferior to 0.0001 suggests that the generated model has a good fit in the whole regression area, and that it is significant from statistical point of view.

The *F*-value of 5.66 denotes that the lack of fit is not significant relative to the pure error. Furthermore, its *p* value of 0.1538 (higher than 0.05) advances the idea that the data predicted by the model are very similar to those obtained through experiments. The *F*-values for sodium hydroxide volume, sodium hydroxide concentration and working temperature were 1307.43, 227.30 and 175.21, showing that the eluent volume has the highest influence on the desorption process. *p*-values less than 0.05 designate that the model terms are significant. In this case, all three factors in linear form and the quadratic forms of NaOH volume and of NaOH concentration are considered significant terms.

The correlation coefficient (R^2^) is 0.9973, while the predicted correlation coefficient (R^2^ predicted) is 0.9601, and the adjusted correlation coefficient (R^2^ adjusted) is 0.9923. Their closeness to 1 and the difference between the last 2 of them being lower than 0.2 sustain the accuracy of the obtained model. The coefficient of variation of 0.7937% also indicates the consistency of the conducted experiments with the predicted data. The adequate precision of 44.3707 (>4) shows a satisfactory signal and supports the conclusion that the model is suitable to navigate the design space.

As mentioned above, the simultaneous optimization of the three factors was carried out with the aim of achieving the highest desorption efficiency of RB from the prepared adsorbent. The subsequent values were the following: NaOH volume: 30 mL; NaOH concentration: 1.5 M; and temperature: 40 °C. Three extra trials were led with these settings. The desorption efficiency was of 86.20 ± 0.33%, highlighting the adequacy of the mathematical model and endorsing it.

### 2.2. Adsorbent Reusability

**Figure 6:**
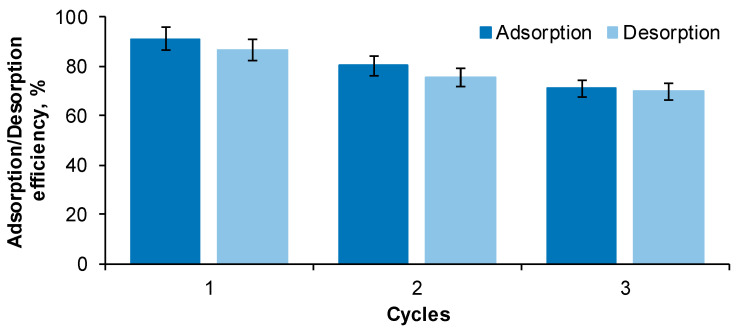
Adsorption/desorption efficiency of Reactive Black 5 dye on/from the prepared adsorbent (Adsorption conditions: RB volume: 30 mL, RB initial concentration: 30 mg/L, adsorbent amount: 3 g, contact time: 6 h, temperature: 30 °C; Desorption conditions: eluent volume: 30 mL, eluent concentration: 1 M, adsorbent amount: 3 g, contact time: 6 h, temperature: 40 °C).

The focal purpose of the regeneration of cherry stones–chitosan hydrogel (CSCH) beads was to establish its ability of adsorbing RB dye after multiple consecutive adsorption/desorption cycles. Three such cycles were performed when the selection of eluent and the optimization of the working conditions by RSM-BBD were finalized. The process evolution for an initial contaminant concentration of 30 mg/L is pictured in Figure 6.

For the first cycle, after 360 min of contact with the RB solution or NaOH eluent, respectively, the adsorption efficiency was of 91.14 ± 0.11% while the desorption reached 86.40 ± 0.24%. In the second cycle, a decline was noted both for the adsorption and for the desorption step (RB removal efficiency was of 80.12 ± 0.30%, while the recovery was of 75.30 ± 0.19%). At the end of the third cycle, the adsorbent was able to retain only 70.98 ± 0.09% of the pollutant from its aqueous solution. Almost the same amount of dye (69.66 ± 0.17%) was desorbed in NaOH medium. The saturation of the active sites existing on the adsorbent can be the probable cause of this behavior.

Other reports on dyes adsorption/desorption reveal a similar possibility of reusability. Three successive cycles were conducted by Hsueh et al. [26] in the research regarding the recyclability of the prepared material for Reactive Black 5 adsorption. Park et al. [30] studied the retention and retrieval of three dyes (Methylene blue, Orange G and Congo red) on/from biochars and discovered that after 4 cycles, the material preserved more than 85% of its adsorption capacity. El Messaoudi et al. [28] used for 4 times the adsorbents based on date stones and jujube shells for Congo red adsorption/desorption with an efficiency higher than 50%.

In relation to the adsorbent characteristics, with the increase in the number of cycles, a slight swelling phenomenon was detected (especially for the desorption phase), and the beads firmness started to decrease.

Explorations by scanning electron microscopy and by Fourier-transform infrared spectroscopy were led in order to analyze the changes appearing during the adsorption/desorption cycles.

**Figure 7:**
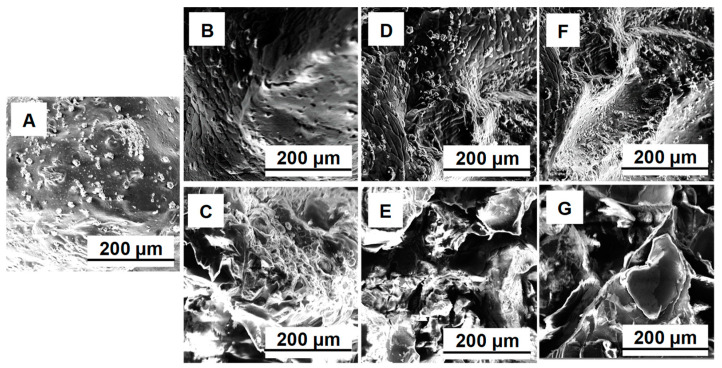
SEM images for the prepared adsorbent before RB adsorption (**A**), after 1st, 2nd and 3rd cycle of RB adsorption (**B**,**D**,**F**) and after 1st, 2nd and 3rd cycle of RB desorption (**C**,**E**,**G**) (Device: TESCAN MIRA (TESCAN Orsay Holding, Brno, Czech Republic); Detection mode: Normal secondary electron in high vacuum; Detector: large field detector; Accelerating voltage: 20 keV; Beam current: 300 pA; Magnification: 320×; Field of view: 873 µm; Working distance: 5 mm; Scan speed: 6).

Figure 7 displays the SEM photographs of the prepared adsorbent and its progress through the process. Initially, CSCH beads present a relatively compact and dense structure (Figure 7A). Some morphological variations appear after the first cycle of adsorption/desorption (Figure 7B,C), and irregularities accentuate after the second cycle (Figure 7D,E). At the end of the third desorption cycle (Figure 7G), the surface presents large and visibly open pores. It can be appreciated that the differences between the adsorption and the desorption can be attributed to the swelling effect observable during desorption, an effect that in the adsorption is reduced by the presence of the acid environment and by the lower temperature required for proper RB retention.

**Figure 8:**
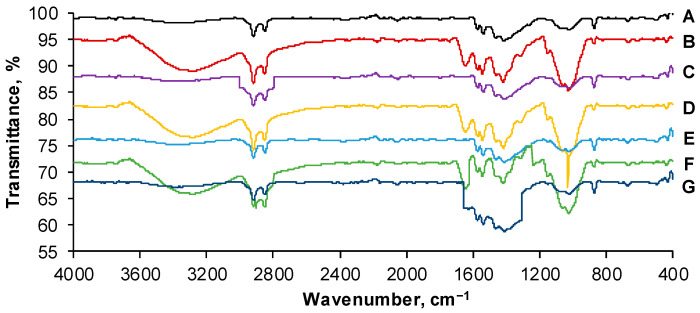
FTIR spectra for CSCH adsorbent before RB adsorption (**A**), after 1st, 2nd and 3rd cycle of RB adsorption (**B**,**D**,**F**) and after 1st, 2nd and 3rd cycle of RB desorption (**C**,**E**,**G**) (Device: IRSpirit FT-IR spectrometer (Shimadzu, Bucharest, Romania); Accessory: QATR; Wavenumber range: 4000 cm^−1^–400 cm^−1^; Scans: 45 scans/min.; Resolution: 4 cm^−1^; Background spectrum reference: air; Cleaning solution: ethanol).

FTIR spectra registered for the adsorbent prepared from cherry stones powder immobilized on chitosan polymer before any adsorption and after Reactive Black 5 dye adsorption/desorption cycles are given in Figure 8.

The polymeric matrix (Figure 8A) is reflected by the presence of the band from 3500 cm^−1^ to 3200 cm^−1^ specific for hydroxyl stretching vibration coinciding with –N–H vibration. The peak of around 2800 cm^−1^ can be assigned to methylene and methylidene asymmetric stretch [31]. Between 1500 cm^−1^ and 1200 cm^−1^, vibrations of –N–H and of –CH_2_ can be detected, while at circa 1000 cm^−1^, vibration of the C–O of the natural polymer or of cherry stones is visible [32]. A high degree of similarity can be distinguished for spectra of the synthesized material after the first and the second cycle of RB desorption (Figure 8C,E).

In the case of the adsorbent loaded with RB dye molecules (Figure 8B,D,F), C–C, C=C and N=N bands typical for benzene and azo stretch from the aromatic structure of the studied pollutant structure were observable from 1600 cm^−1^ to 1450 cm^−1^ [33,34]. Between 1100 cm^−1^ and 1000 cm^−1^ stretches of –S=O– and of sulfoxide existing in RB can be recognized [21].

The peaks intensity differs from an adsorption/desorption cycle to another, and some degradation of the adsorbent beads integrity is easily noticeable in the region of 1200 cm^−1^ to 1700 cm^−1^ after the third desorption (Figure 8G).

### 2.3. Impact of Contact Time on Reactive Black 5 Desorption. Kinetic Studies

One of the most important factors influencing the adsorption process of contaminants from aqueous solutions is represented by the time of contact between the adsorbate and the adsorbent. Our anterior results [19] as those of other studies dedicated to the preparation of adsorbing materials and to their applications [35,36,37,38] assert that the RB adsorption follows closely the pseudo-second-order kinetics.

It is expected that contact time has a major effect on the desorption step as well.

In order to evaluate the consequences of the interaction period, 3 g of CSCH loaded with RB from aqueous solutions (having concentrations of 10 mg/L, 20 mg/L, 30 mg/L, 40 mg/L and 50 mg/L) were immersed in 30 mL of NaOH 1.5 M at a temperature of 40 °C. The desorption evolution was followed between 15 min and 360 min during 3 different cycles.

The recovered data were fitted into two different kinetic models: pseudo-first-order model (PS1) and pseudo-second-order model (PS2), both modified for desorption. The amount of dye that remained on the adsorbent sites after elution was considered as rate-limiting step [39]. PS1 adjusted for desorption stipulates that the desorption rate is related with the number of sites occupied with RB molecules, while for modified PS2, the desorption rate is proportional to the squared number of sites charged with RB.

**Figure 9:**
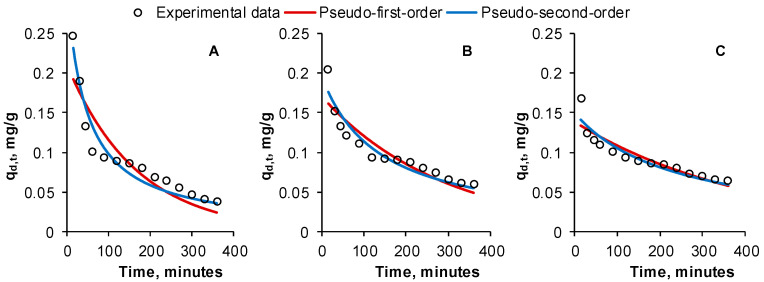
Desorption kinetics of Reactive Black 5 dye from the prepared adsorbent during the first cycle (**A**), second cycle (**B**) and third cycle (**C**) (RB volume: 30 mL, RB initial concentration: 30 mg/L, eluent volume: 30 mL, eluent concentration: 1 M, adsorbent amount: 3 g, contact time: 6 h, temperature: 40 °C).

For all five initial concentrations existing in RB solutions, the kinetic was similar. Thus, we have pictured in Figure 9 only the allures obtained for the case of using RB solutions with a concentration of 30 mg/L.

It can be noted that in all 3 desorption cycles, in the first 60 min, the elution of RB from the adsorbent is rapid, and then the rate slows down. This can be explained by the high concentration of dye existing in the prepared material at the desorption start. The clean eluent also favors the migration of the adsorbate due to the concentration difference. In time, the desorbent enriches in RB and the elution reduces until equilibrium.

Parameters of the applied kinetic models are given in Table 3. Their analysis shows that the PS2 model is more suitable for fitting the experimental results than PS1. Our outcomes are comparable with those reported by Bhatti et al. [40] who used rice husk powder modified through physico-chemical treatments as adsorbent for four different dyes from aqueous solutions. The desorption followed a two-step kinetic model. In their study on the adsorption–desorption of methylene blue with bentonite adsorbent coating, Momina et al. [41] established, too, that PS2 kinetic adequately describes the relation between the desorbent and the tested dye. PS2 was also the best model for kinetic of Congo red desorption from a composite prepared by Goddeti et al. [42].

Various statistical functions were employed to establish the mathematical models accuracy. Sum of square error (SSE) represents the total value of the square difference calculated between the experimental and predicted data. Mean square error (MSE) is established by dividing SSE to the number of the conducted experiments. Root mean square error (RMSE) scrutinizes the models with ideal magnitude. Average relative error (ARE) tries to diminish the error distribution through the whole studied concentration range. Chi-square (χ^2^) compares the observed results with those predicted by the model. The correlation coefficient (R^2^) measures the data estimated to fit the regression line and reveals if the variations of the dependent variables are related to those of independent ones. Values for these error functions determined for the tested kinetic models are included in Table 3 for all three desorption cycles. The smaller results of SSE, MSE, RMSE, ARE and χ^2^ along with the higher results for R^2^ also reveal that PS2 is more adequate than PS1 for describing the kinetics of RB desorption from the synthesized adsorbent.

### 2.4. Influence of Reactive Black 5 Initial Concentration on Desorption. Equilibrium Isotherms

It is common practice to use isotherms in order to establish the interactions occurring between the contaminant from an aqueous solution and the material used for removing it by adsorption. Regarding their application in the case of dye desorption from the loaded adsorbent to a liquid eluent, the studies are not so frequent.

Thus, one of the aims of our present work was to examine the possibility of applying isotherm models for the desorption of RB from CSCH by elution with sodium hydroxide. Two equilibrium isotherms with two parameters (Langmuir and Freundlich) and three with three parameters (Redlich–Peterson, Sips and Toth) were selected and fitted to the acquired experimental data.

Originally employed to measure gases retention on solid support [43], the Langmuir isotherm relies on the facts that the adsorbent has a homogenous surface, the adsorbate does not interact with other active sites except those it occupies, and the desorption is monolayer and irreversible. Langmuir has as characteristic a plateau attainable at important concentrations [44].

Opposing, the Freundlich isotherm assumes that the desorption is reversible. It considers that the adsorbent has a heterogeneous surface, and that there are interactions between the adsorbed molecules [45]. It describes accurately only experiments carried out in mild concentration range and lacks essential thermodynamic foundation [46].

Redlich–Peterson is a combination of the above mentioned two isotherms and does not discriminate the adsorbents based on their homogeneity. At reduced concentrations, it moves toward Henry’s law, while at elevated concentrations, it becomes similar to the Freundlich model [47].

Also called the Langmuir–Freundlich isotherm, the Sips model is similar to the Freundlich equation when the pollutant concentration is reduced and comparable to the Langmuir expression in the case of elevated concentrations [48].

The Toth equilibrium isotherm modifies the Langmuir characteristics for achieving a diminished error between the calculated values and the observed ones. It is applied both at reduced and elevated adsorbate concentrations and considers the adsorbent surface as heterogeneous. The model presents asymmetrical distribution of energy, suggesting that the sorption energy of the active sites of an adsorbent is lower than the mean value [49].

In one of our already published papers [19], we revealed that the Langmuir model was not suitable to describe the RB adsorption behavior on the prepared adsorbent material. As can be remarked, nor can the desorption process be described by this isotherm. The maximum desorption capacities predicted are significantly higher than those obtained through experiments (0.236 ± 0.022 mg/g for the first desorption cycle, 0.181 ± 0.016 mg/g for the second desorption cycle, 0.148 ± 0.014 mg/g for the last desorption cycle).

**Figure 10:**
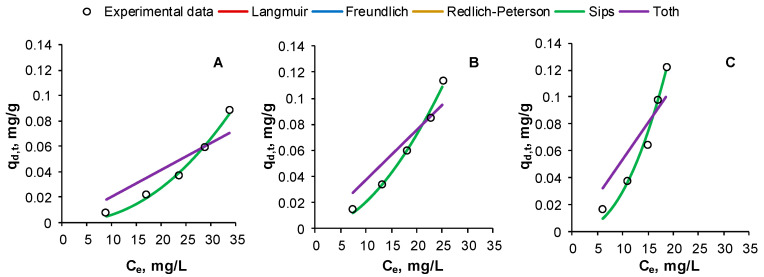
Equilibrium isotherms for Reactive Black 5 dye desorption from the prepared adsorbent in first cycle (**A**), second cycle (**B**) and third cycle (**C**) (RB volume: 30 mL, RB initial concentration: 30 mg/L, eluent volume: 30 mL, eluent concentration: 1 M, adsorbent amount: 3 g, contact time: 6 h, temperature: 40 °C).

The graphical representation of experimental data fitted on the cited isotherm models and the specific calculated parameters are visible in Figure 10 and in Table 4 for the three desorption cycles. Overlaps of the Langmuir, Redlich–Peterson and Toth isotherms and of the Freundlich and Sips models can be mentioned.

Alike inference is valid for all isotherms with three-parameters tested. Even though the Sips model seems to portray the measured data, a closer look on its parameters also reveals a maximum desorption capacity much different from the real one. With respect to the Redlich–Peterson and Toth isotherms, the discrepancy between the experimental and the predicted values can be easily observed. Our investigation shows that the Freundlich model is more appropriate to follow the input values, suggesting that CSCH is an adsorbent possessing a heterogeneous surface, and that the desorption of the emergent compound is a multilayer type. Our deduction is supported by the results of statistical functions employed for validating the mathematical models. Table 4 indicates that Freundlich has the lowest values of SSE, MSE, RMSE, ARE and χ^2^ recorded for the three desorption cycles and the highest correlation coefficients (0.9911 for 1st desorption cycle, 0.9884 for 2nd desorption cycle and 0.9847 for 3rd desorption cycle).

As explained earlier, the suppleness of the Freundlich equation makes it suitable for the interpretation of desorption equilibrium in an extensive range of concentrations from adsorbents having homogeneous and heterogeneous surfaces. In their research, Momina et al. [41] showed likewise that the desorption of methylene blue is governed by Freundlich model, but, opposing to our conclusion, in their case, the Sips isotherm appears to be adequate too. Ahammad et al. [50] report the sequence Redlich–Peterson > Freundlich > Sips > Langmuir for the order of isotherms able to follow the experimental data obtained for the desorption of another water contaminant.

## 3. Conclusions

The current research was focused on analyzing the desorption of a model azo dye molecule (Reactive Black 5) from a material with adsorbing properties obtained by immobilizing cherry stones powder on a chitosan matrix.

Water, one base (sodium hydroxide), one acid (hydrochloric acid), one salt (sodium chloride) and one alcohol (ethanol) were chosen as possible desorbents. Among them, sodium hydroxide was suitable for eluting the retained compound from the prepared material. A Response Surface Methodology-Box–Behnken Design served to establish that with a volume of 30 mL of NaOH having a concentration of 1.5 M at a temperature of 40 °C after 6 h, the desorption efficiency is superior to 85%. After three successive cycles of adsorption/desorption, 69.66% of the dye load were eluted from the adsorbent.

Pseudo-second-order kinetic and Freundlich equilibrium isotherm described with high accuracy the desorption process, the associated correlation coefficients being very close to unit.

Our results sustain the fact that the synthesized material is adequate for adsorbing the target molecule and can efficaciously be reused multiple times.

Further investigations encompassing a more advanced optimization of the desorption conditions, the test of other kinetic and isotherm models and the application on multiple refractory compounds existing in a real aqueous matrix are in progress.

## 4. Materials and Methods

All experiments were carried out in duplicate and reported as mean values.

### 4.1. Chemical Reagents

Only reagents of analytical grade with no applied pretreatment and distilled water were used to prepare stock solutions and their subsequent dilutions.

Chemical Company (Iasi, Romania) delivered sodium chloride, sodium hydroxide, hydrochloric acid, acetic acid, methanol and ethanol. Glutaraldehyde was purchased from Across Organics (Vienna, Austria). Merck (Bucharest, Romania) provided potassium nitrate, chitosan and Reactive Black 5 dye.

### 4.2. Analytical Procedure

A 100 mg/L stock solution of Reactive Black 5 was prepared with distilled water and diluted as required for the experimental program.

When needed, pH corrections were made with reduced volumes of NaOH 0.1 M or of HCl 0.1 M. A pH tester HI 98103 from Hanna Instruments (Bucharest, Romania) served for pH measurements.

A calibration curve with RB concentrations between 1 mg/L and 40 mg/L was plotted after reading the absorbance at the wavelength of 600 nm with the help of a UV1280 Spectrophotometer (Shimadzu, Kyoto, Japan).

### 4.3. Batch Adsorption–Desorption of Reactive Black 5 Dye

The adsorbent used for removing RB from aqueous media was represented by cherry stones–chitosan hydrogel beads (CSCH) which were prepared according to the methodology exposed by Altun et al., 2019 [32] with some amendments [19]. Succinctly, 4 g of chitosan were submerged in 200 mL of a 2% acetic acid solution, and an intensive mixing was realized on a Nahita magnetic plate (Auxilab, Beriáin, Spain). Then, 2 g of cherry stone powder were added, and the stirring was continued for 24 h. The resultant mixture was dropped into a coagulation solution formed of 120 g of NaOH, 400 mL of distilled water and 600 mL of methanol. The obtained hydrogel beads were maintained in this solution for 24 h until complete coagulation. They were then washed with distilled water up to neutral pH. They were introduced in a flat bottom laboratory balloon along with a solution containing 1.2 mL glutaraldehyde and 120 mL methanol and heated at reflux at 70 °C for 6 h. The prepared hydrogel beads (CSCH) were stored in a glutaraldehyde–methanol solution in a closed vessel at 4 °C. Before use, they were washed with ethanol and distilled water.

The adsorption was directed for 6 h at 30 °C. CSCH beads and RB solution, having a concentration of 30 mg/L and pH 2, were introduced in an Erlenmeyer flask in a ratio of 1:1 (*w*/*v*). The absorbance of the solution after adsorption was read at 600 nm, and the final concentration was established against the calibration curve.

Adsorption removal efficiency (R, %) and adsorption equilibrium capacity (q_e, ads._, mg/g) were acquired with Equations (2) and (3):

(2) $$R=\frac{C_{i}-C_{e}}{C_{i}}\times 100$$

(3) $$q_{e,\;\;ads.}=\frac{C_{i}-C_{e}}{m}\times V$$

wherein *C_i_* and *C_e_* are the initial and at equilibrium concentrations (mg/L); *V* is RB dye volume (L), and *m* is the adsorbent mass (g).

For the desorption study, five different solvents, namely distilled water, NaCl 1 M, NaOH 1 M, HCl 1 M and ethanol 1 M, were used. The same conditions as for adsorption were considered with reference to time, temperature and solid–liquid ratio as well as for establishing the final concentration existing in the solution.

Desorption capacity (*q_d_*_,*t*,*des*._, mg/g) and desorption efficiency (*D*, %) were calculated with Equations (4) and (5):

(4) $$q_{d,t,\;des.}=\frac{C_{f}}{m}\times V$$

(5) $$D=\frac{q_{d,t,\;des.}}{q_{e,\;ads.}}\times 100$$

where *C_f_* is the RB final concentration in the solvent (mg/L); *V* is the solvent volume (L); *m* is the adsorbent amount (g); *q_d,t,des._* is the desorption capacity (mg/g); and *q_e,ads._* is the adsorption capacity (mg/g).

Once the adequate desorbent was chosen, a Response Surface Methodology-Box–Behnken Design (BBD) with three factors and three levels of variation was applied for the investigation and validation of desorption parameters. RB initial concentration was kept constant at 30 mg/L. The desorbent volume (10–30 mL), its concentration (0.5–1.5 M) and the working temperature (20–40 °C) were the input variables. Their levels were coded as low (−1), medium (0) and high (+1). The design matrix is detailed in Table 1.

Design Expert 13 software (Stat-Ease, Minneapolis, MN, USA) was used to optimize the input factors and to obtain a second order polynomial model whose general form is given by Equation (6).

(6) $$Y=\alpha_{0}+\alpha_{1}\times A+\alpha_{2}\times B+\alpha_{3}\times C+\alpha_{4}\times A\times B+\alpha_{5}\times A\times C+\alpha_{6}\times B\times C+\alpha_{7}\times A^{2}+\alpha_{8}\times B^{2}+\alpha_{9}\times C^{2}$$

where *Y* is the response function (desorption efficiency, %), *A*, *B* and *C* are independent variables (NaOH volume, NaOH concentration and working temperature respectively), *α*_0_ is the intercept, *α*_1_, *α*_2_ and *α*_3_ are linear coefficients, *α*_4_, *α*_5_ and *α*_6_ are quadratic coefficients, and *α*_7_, *α*_8_ and *α*_9_ are interaction coefficients.

### 4.4. Adsorbent Reusability

Three successive cycles of adsorption–desorption were performed. For the adsorption step, 30 mL of RB with initial concentrations between 10 mg/L and 50 mg/L and 3 g of CSCH beads were used. For the desorption phase, the optimal conditions resulted from BBD were employed. The amount of the pollutant remained on the adsorbent material was followed from 15 min to 240 min.

### 4.5. Adsorbent Characterization

SEM analysis and FTIR spectra of CSCH before and after the adsorption–desorption were carried out in the same conditions as presented in our previous article [19].

For SEM investigation, a TESCAN MIRA device (TESCAN Orsay Holding, Brno, Czech Republic) equipped with TESCAN Essence software version 1.0.8.0 was employed. The adsorbent beads were firstly dried for 12 h at ambient temperature. Double-adhesive carbon discs served to fix the material on the specific microscope stubs. The specific parameters were set as follows: detection mode—normal secondary electron in high vacuum; detector—large field detector; accelerating voltage—20 keV; beam current—300 pA; magnification—320×; field of view—873 µm; working distance—5 mm; scan speed—6.

IRSpirit FT-IR spectrometer (Shimadzu, Bucharest, Romania) including a QATR accessory served for FTIR spectra recording. The wavenumber range was between 4000 cm^−1^ and 400 cm^−1^ with 45 scans/min and a resolution of 4 cm^−1^. The cleaning of the QATR plate after each spectrum acquisition was carried out with ethanol. The background spectrum reference was that of air.

### 4.6. Desorption Kinetic and Equilibrium Isotherms

Several nonlinear kinetic and equilibrium isotherm models (Table 5 and Table 6) were applied to the obtained desorption data. OriginPro 2019b software (OriginLab, Northampton, MA, USA) and CAVS software, version 2.0 (Federal University of Paraná, Curitiba, Paraná, Brazil) were used for records treatment.

### 4.7. Statistical Analysis

The experimental values were evaluated from a statistical point of view by Analysis of Variance (ANOVA) (in the case of RSM-BBD) and by sum of square error (*SSE*)—Equation (7), mean squared error (*MSE*)—Equation (8), root mean square error (*RMSE*)—Equation (9), chi-square (χ^2^)—Equation (10), average relative error (*ARE*)—Equation (11) and coefficient of determination (*R*^2^)—Equation (12) (in the case of the kinetic and equilibrium studies).

(7) $$SSE=\overset{n}{\underset{i=1}{\sum }}{\left(q_{d,t,\;exp.}-q_{d,t,pred.}\right)}^{2}$$

(8) $$MSE=\frac{\sum_{i=1}^{n}{\left(q_{d,t,\;exp.}-q_{d,t,pred.}\right)}^{2}}{n}$$

(9) $$RMSE=\sqrt{\frac{\sum_{i=1}^{n}{\left(q_{d,t,\;exp.}-q_{d,t,pred.}\right)}^{2}}{n-2}}$$

(10) $$ARE=\frac{100}{n}\cdot \overset{n}{\underset{i=1}{\sum }}\left|\frac{q_{d,t,\;exp.}-q_{d,t,pred.}}{q_{d,t,\;exp.}}\right|$$

(11) $$\chi^{2}=\overset{n}{\underset{i=1}{\sum }}\frac{{\left(q_{d,t,\;exp.}-q_{d,t,pred.}\right)}^{2}}{q_{d,t,pred.}}$$

(12) $$R^{2}=1-\frac{\sum_{i=1}^{n}{\left(q_{d,t,exp.}-q_{d,t,pred.}\right)}_{i}^{2}}{\sum_{i=1}^{n}{\left(q_{d,t,exp.}-{\bar{q}}_{d,t,exp.}\right)}_{i}^{2}}$$

wherein *q_d_*_,*t*,*exp.*_ and *q_d_*_,*t*,*pred.*_ are the experimental and predicted values of pollutant remained in adsorbent after desorption; ${\bar{q}}_{d,t,exp.}$ is the average of measured values of *q_d_*_,*t*,*exp.*_, and *n* is the number of observations in experimental data.

## Figures and Tables

**Table 1:** RSM-BBD matrix comprising experimentally acquired and model-predicted records for the desorption of Reactive Black 5 dye from the prepared adsorbent.

Run	Variables—Actual and Coded Values			Desorption Efficiency, %	
	A	B	C	Observed	Predicted
1	10 (−1)	0.5 (−1)	30 (0)	62.70	63.06
2	30 (+1)	0.5 (−1)	30 (0)	78.17	78.13
3	20 (0)	1.5 (+1)	40 (+1)	82.89	82.68
4	20 (0)	1.5 (+1)	20 (−1)	76.01	76.54
5	30 (+1)	1.5 (+1)	30 (0)	85.10	84.74
6	10 (−1)	1 (0)	20 (−1)	64.01	63.44
7	10 (−1)	1 (0)	40 (+1)	69.47	69.63
8	30 (+1)	1 (0)	40 (+1)	83.77	84.34
9	20 (0)	0.5 (−1)	40 (+1)	76.29	75.76
10	20 (0)	1 (0)	30 (0)	77.87	77.77
11	30 (+1)	1 (0)	20 (−1)	79.50	79.33
12	20 (0)	1 (0)	30 (0)	78.02	77.77
13	20 (0)	1 (0)	30 (0)	77.43	77.77
14	10 (−1)	1.5 (+1)	30 (0)	69.17	69.21
15	20 (0)	0.5 (−1)	20 (−1)	70.50	70.70

**Table 2:** ANOVA for Reactive Black 5 dye from the prepared adsorbent.

Source	Sum of Squares	df	Mean Square	*F*-Value	*p*-Value
Model	650.40	9	72.27	201.82	<0.0001
A—NaOH volume	468.15	1	468.15	1307.43	<0.0001
B—NaOH concentration	81.39	1	81.39	227.30	<0.0001
C—Temperature	62.74	1	62.74	175.21	<0.0001
AB	0.0538	1	0.0538	0.1503	0.7142
AC	0.3493	1	0.3493	0.9755	0.3687
BC	0.2943	1	0.2943	0.8219	0.4062
A^2^	35.78	1	35.78	99.93	0.0002
B^2^	2.84	1	2.84	7.93	0.0373
C^2^	0.8376	1	0.8376	2.34	0.1867
Residual	1.79	5	0.3581		
Lack of Fit	1.60	3	0.5339	5.66	0.1538
Pure Error	0.1887	2	0.0943		
Cor Total	652.19	14			

**Table 3:** Kinetic parameters and statistical functions of Reactive Black 5 dye desorption from the prepared adsorbent.

Kinetic Model	Parameters	Values		
		1st Desorption Cycle	2nd Desorption Cycle	3rd Desorption Cycle
Pseudo-first-order	*q_d_*_,1_	0.2103	0.1701	0.1387
	*k_d_*_,1_	0.0059	0.0034	0.0024
	SSE	0.007940	0.003271	0.001710
	MSE	0.000567	0.000234	0.000122
	RMSE	0.000662	0.000273	0.000142
	ARE	21.151430	10.657220	7.542603
	χ^2^	0.005771	0.025682	0.014560
	R^2^	0.8228	0.8428	0.8294
Pseudo-second-order	*q_d_*_,2_	0.3051	0.1959	0.1497
	*k_d_*_,2_	0.0700	0.0369	0.0279
	SSE	0.003016	0.001788	0.001218
	MSE	0.000215	0.000128	0.000087
	RMSE	0.000251	0.000149	0.000101
	ARE	14.008901	8.579999	6.843968
	χ^2^	0.032073	0.014693	0.010110
	R^2^	0.9333	0.9110	0.8784

**Table 4:** Equilibrium isotherm parameters and statistical functions of RB desorption from CSCH.

Isotherm	Parameters	Values		
		1st Desorption Cycle	2nd Desorption Cycle	3rd Desorption Cycle
Langmuir	*Q_L_*	3248.818	3286.657	3258.273
	*K_L_*	6.394 × 10^−7^	1.184 × 10^−6^	1.643 × 10^−6^
	SSE	0.000752	0.000846	0.001425
	MSE	0.000150	0.000169	0.000285
	RMSE	0.000251	0.000282	0.000475
	ARE	49.542003	32.758021	40.071366
	χ^2^	0.000762	0.000843	0.001425
	R^2^	0.8130	0.8645	0.8073
Freundlich	*K_F_*	4.141 × 10^−5^	3.025 × 10^−4^	1.832 × 10^−4^
	*n*	0.460	0.547	0.451
	SSE	0.000035	0.000072	0.000113
	MSE	0.000007	0.000014	0.000022
	RMSE	0.000019	0.000024	0.000037
	ARE	13.420319	7.524428	12.111494
	χ^2^	0.000033	0.000072	0.000114
	R^2^	0.9911	0.9884	0.9847
Redlich–Peterson	*K_RP1_*	0.019	0.043	0.056
	*K_RP2_*	8.255	10.413	9.468
	*n_RP_*	4.079 × 10^−15^	5.724 × 10^−17^	1.379 × 10^−15^
	SSE	0.000752	0.000845	0.00145
	MSE	0.000150	0.000169	0.000285
	RMSE	0.000251	0.000282	0.000475
	ARE	49.543761	32.742932	40.072206
	χ^2^	0.001125	0.001266	0.002136
	R^2^	0.8130	0.8645	0.8073
Sips	*Q_S_*	101,770.961	8841.882	78,576.519
	*K_S_*	4.806 × 10^−5^	8.274 × 10^−5^	1.294 × 10^−4^
	*n_S_*	2.175	1.829	2.221
	SSE	0.000035	0.000072	0.000113
	MSE	0.000007	0.000014	0.000022
	RMSE	0.000011	0.000024	0.000037
	ARE	13.427219	7.553481	12.150279
	χ^2^	0.000051	0.000108	0.000168
	R^2^	0.9911	0.9884	0.9847
Toth	*Q_TO_*	5.918	6.053	6.548
	*K_TO_*	0.003	0.005	0.007
	*n_TO_*	10.302	9.065	8.778
	SSE	0.000753	0.000849	0.001425
	MSE	0.000151	0.000170	0.000285
	RMSE	0.000251	0.000283	0.000475
	ARE	49.572085	32.822459	40.072203
	χ^2^	0.001125	0.002535	0.002136
	R^2^	0.8130	0.8645	0.8073

**Table 5:** Desorption kinetic models equations.

Kinetic Model	Equation	Parameters Significance
Pseudo-1st-order	$q_{d,t}=\frac{q_{d,1}}{e^{k_{d,1}\cdot t}}$	*k_d_,*_1_—pseudo-first-order constant rate, min^−1^
Pseudo-2nd-order	$q_{d,t}=\frac{k_{d,2}}{1+k_{d,2}\cdot q_{d,2}\cdot t}$	*k_d,_*_2_—pseudo-2nd-order constant rate, g/(mg·min)

*q_d_*_,*t*_—concentration on the solid phase at time *t*, mg/g; *q*_*d*,1_, *q*_*d*,2_ —adsorbent capacity at equilibrium, mg/g; *t*—contact time, min.

**Table 6:** Desorption equilibrium isotherms nonlinear equations.

Equilibrium Isotherm	Equation	Parameters Significance
Langmuir	$q_{d,t}=\frac{Q_{L}\cdot K_{L}\cdot C_{e}}{1+K_{L}\cdot C_{e}}$	*Q_L_*—Langmuir maximum uptake, mg/g *K_L_*—Langmuir constant, L/mg
Freundlich	$q_{d,t}=K_{F}\cdot C_{e}^{1/n}$	*K_F_*—Freundlich constant, (mg/g) (L/mg)^1/*n*^ *n*—Freundlich constant, dimensionless
Redlich–Peterson	$q_{d,t}=\frac{K_{RP1}\cdot C_{e}}{1+K_{RP2}\cdot C_{e}^{n_{RP}}}$	*K_RP1_*—Redlich–Peterson constant, L/g *K_RP2_*—Redlich–Peterson constant, L/mg *n_RP_*—Redlich–Peterson constant, dimensionless
Sips	$q_{d,t}=\frac{Q_{S}\cdot {\left(K_{S}\cdot C_{e}\right)}^{n_{S}}}{1+{\left(K_{S}\cdot C_{e}\right)}^{n_{S}}}$	*Q_S_*—Sips maximum uptake, mg/g *K_S_*—Sips constant, L/mg *n_S_—*Sips constant, dimensionless
Toth	$q_{d,t}=\frac{Q_{To}\cdot C_{e}}{{\left(a_{To}+C_{e}\right)}^{1/n_{To}}}$	*Q_To_*—Toth maximum uptake, mg/g *K_To_*—Toth constant, L/mg *n_To_—*Toth constant, dimensionless

*q_d_*_,*t*_—adsorbate concentration on solid phase at equilibrium, mg/g; *C_e_*—adsorbate concentration on fluid phase at equilibrium, mg/L.

## Data Availability

Not applicable.
